# Supplementary material for: FORUM: building a Knowledge Graph from public databases and scientific literature to extract associations between chemicals and diseases
Source: Bioinformatics. 2021 Sep 3;37(21):3896–904. doi: 10.1093/bioinformatics/btab627 (PMC8570811; doi:10.1093/bioinformatics/btab627)
Supplement: btab627_Supplementary_Data [file btab627_supplementary_data.pdf]

FORUM: Building a Knowledge Graph from public databases and scientific literature to extract associations between chemicals and diseases.

M. Delmas[1], O. Filangi[2], N. Paulhe[3], F. Vinson[1], C. Duperier[3], W. Garrier[4], P.-E. Saunier[4], Y. Pitarch[5], F. Jourdan[1], F. Giacomoni[3], and C. Frainay[1]

[1] *Toxalim (Research Center in Food Toxicology), Université de Toulouse, INRAE, ENVT, INP-Purpan, UPS, 31300 Toulouse, France*
[2] *IGEPP, INRAE, Institut Agro, Université de Rennes, Domaine de la Motte, 35653 Le Rheu, France*
[3] *Université Clermont Auvergne, INRAE, UNH, Plateforme d'Exploration du Métabolisme, MetaboHUB Clermont, F-63000 Clermont-Ferrand, France*
[4] *ISIMA, Campus des Cézeaux, 63177 Aubière, France*
[5] *IRIT, Université de Toulouse, Cours Rose Dieng-Kuntz, 31400 Toulouse, France*

S1 Supplementary tables

RANK	MESH_LABEL	MESH	PubMed Articles	q-value	odds-ratio	chisq-stat
1	Pseudohypoparathyroidism	D011547	234	1.30e-277	48.38	7749
2	Neuroblastoma	D009447	662	1.02e-260	5.76	2463
3	Neuroectodermal Tumors, Primitive, Peripheral	D018241	666	1.50e-260	5.72	2452
4	Neuroectodermal Tumors, Primitive	D018242	675	1.56e-239	5.15	2141
5	Neoplasms, Neuroepithelial	D018302	1082	1.17e-184	2.86	1258
6	Neoplasms, Germ Cell and Embryonal	D009373	1786	8.33e-171	2.13	1026
7	Neuroectodermal Tumors	D017599	1637	8.07e-163	2.17	985
8	Neoplasms, Nerve Tissue	D009380	1662	1.43e-159	2.13	959
9	Cystic Fibrosis	D003550	365	4.90e-98	3.95	772
10	Leydig Cell Tumor	D007984	114	1.04e-94	18.54	1628
11	Sertoli-Leydig Cell Tumor	D018310	117	1.51e-86	14.60	1311
12	Parathyroid Diseases	D010279	429	4.44e-82	3.06	576
13	Cholera	D002771	125	8,62e-80	11.17	1050
14	Graves Disease	D006111	249	3,97e-77	4.48	643
15	Calcium Metabolism Disorders	D002128	606	3.05e-74	2.37	466
16	Sex Cord-Gonadal Stromal Tumors	D018312	126	1.40e-72	9.46	875
17	Neoplasms, Gonadal Tissue	D018309	126	2.55e-71	9.20	848
18	Exophthalmos	D005094	249	5.34e-69	4.05	548
19	Polycystic Kidney Diseases	D007690	133	3.11e-64	7.34	680
20	Adrenal Gland Neoplasms	D000310	293	1.18e-63	3.34	463
23	Glioma	D005910	552	2.16e-59	2.23	363
26	Hypoparathyroidism	D007011	126	1.11e-52	6.12	508
27	Hyperparathyroidism	D006961	307	2.29e-51	2.80	345
32	Cell Transformation, Neoplastic	D002471	459	6.72e-46	2.15	276
34	Hypercalcemia	D006934	207	5.31e-42	3.17	297
38	Adrenal Cortex Neoplasms	D000306	109	2.44e-38	5.04	335
42	Osteosarcoma	D012516	199	8.89e-33	2.77	217

Table S1: Top 20 hits of the FORUM search for compound "Cyclic AMP", only considering disease descriptors. Rows in grey represent diseases which are in the reference set but which are ranked below the top 20 in this list. Rows in green represent newly associated MeSH descriptors and those in white are descriptors of the reference set also found in our top 20.

RANK	Metabolite	CID	PubMed Articles	P-Value adj	FoldChange	chisq-Stat
1	L-phenylalanine	6140	2279	0	222.1	499735.3
2	endophenyl	6925665	2279	0	222.1	499735.3
3	D-phenylalanine	71567	2279	0	222.1	499735.3
	DL-Phenylalanine	994	2279	0	219.8	499735.0
5	Sapropterin	44257	278	0	296.4	80864.7
6	AC1L9H5R	444951	278	0	296.4	80864.7
7	6,7-Dihydrobiopterin	133246	278	0	296.2	80918.0
8	7-tetrahydrobiopterin	1125	278	0	295.9	80824.7
9	1-(2-amino-4-hydroxy-5,6,7,8-tetrahydropteridin-7-yl) propane-1,2-diol	169715	278	0	295.9	80824.7
10	Pterin H B2	2380	353	0	206.0	71398.0
11	biopterin	444475	353	0	206.0	71398.0
12	7,8-Dihydrobiopterin	252	353	0	200.3	69422.2
13	L-tyrosine	6057	538	0	28.0	14032.7
14	D-Tyrosine	71098	538	0	28.0	14032.7
15	DL-Tyrosine	1153	538	0	28.0	14032.7
16	2-amino-3-(4-hydroxyphenyl)propanote	5460807	538	0	28.0	14032.7
17	(2S)-2-Azaniumyl-3-(4-hydroxyphenyl)propanoate	6942100	538	0	28.0	14032.7
18	(2S)-2-amino-3-(4-hydroxyphenyl)propanoate	5460822	538	0	28.0	14032.7
19	(2R)-2-amino-3-(4-hydroxyphenyl)propanoate	5460814	538	0	28.0	14032.7
20	LS-188017	24848110	542	0	24.3	12107.4
21	Phenylpyruvic acid	997	39	2.07e-85	339.4	13159.0
22	Enol-phenylpyruvate	641637	39	2.07e-85	339.4	13159.0
23	2-hydroxy-3-phenylprop-2-enoic acid	691	39	2.07e-85	339.4	13159.0
24	Tryptophan	1148	120	3.72e-67	8.9	841.6
25	L-Tryptophan-beta-14C	148495	120	3.72e-67	8.9	841.6

Table S2: Reference set of compounds related to phenylketonurias, from the Top 25 hits of Metab2MeSH search. Rows in red represent compounds which are not found using our knowledge base.

RANK	Metabolite	CID	PubMed Articles	q-value	odds-ratio	chisq-stat
1	(2S)-2-Azaniumyl-3-phenylpropanoate	6925665	2744	0	899.6	717859.4
2	Phenylalanine	6140	3045	0	949.4	695241.7
3	2-Amino-6-[(1S,2R)-1,2-dihydroxypropyl]-5,6,7,8-tetrahydropteridine-4(1H)-one	136153088	436	0	567.0	178498.9
4	Tetrahydrobiopterin	135402045 (eq. of 1125)	436	0	567.0	178498.9
5	5,6,7,8-Tetrahydrobiopterin	135409384	436	0	567.0	178498.9
6	Sapropterin dihydrochloride	135409471	436	0	567.0	178498.9
7	Trihydroxybutyrophenone	129630809	436	0	567.0	178498.9
8	Sapropterin	135398654 (eq. of 44257)	436	0	566.6	178420.0
9	Tetrahydrodictyopterin	135433600	436	0	566.3	178341.3
10	2,4,5-Trihydroxybutyrophenone	15008	436	0	564.0	177791.9
11	D-Erythro-Biopterin	135449517	517	0	390.9	150772.5
12	Orinapterin	135738580	517	0	390.9	150772.5
13	Biopterin	135403659 (eq. of 444475)	517	0	390.9	150772.5
14	d-Threo biopterin	135909519	517	0	390.9	150772.5
15	Pterin H B2	135398729 (eq. of 2380)	521	0	381.0	148652.0
16	4(1H)-Pteridinone,2-amino-7-(1,2-dihydroxypropyl)-5,6,7,8-tetrahydro-	135616732 (eq. of 169715)	195	0	558.4	82872.6
17	(6S)-2-Amino-6-[(1S,2S)-1,2-dihydroxypropyl]-5,6,7,8-tetrahydro-3H-pteridin-4-one	136003108	184	0	361.1	54034.9
18	L-Tyrosinate(1-)	5460822	597	0	35.6	16907.2
19	(2S)-2-Azaniumyl-3-(4-hydroxyphenyl)propanoate	6942100	597	0	35.6	16907.2
20	L-Tyrosine	6057	654	0	16.2	7797.9
21	Phenylpyruvic acid	997	62	1.33e-128	350.5	18030.0
22	Sodium phenylpyruvate monohydrate	23666336	57	2.26e-127	519.6	23312.9
23	Sodium phenylpyruvate	23667645	57	2.26e-127	519.6	23312.9
24	2-Oxo-3-phenylpropanoate	4592697	57	2.26e-127	519.6	23312.9
25	L-Tryptophan	6305	141	2.87e-72	7.9	807.6
30	DL-Tryptophan	1148	124	4.23e-70	9.0	846.8
58	Dihydrobiopterin	135398687 (eq. of 252)	11	4,09e-20	184.8	1684,4

Table S3: Top 25 hits of the FORUM search for the disease group Phenylketonurias (MeSH D010661). Rows in grey represent compounds which are in the reference set but which are ranked lower in this list. ows in green represent newly associated compounds and those in white are compounds of the reference set also found in our list. Some entries in the Metab2MeSH results correspond to old PubChem identifiers, so we looked for equivalences in current PubChem entries. We annotated them with *eq. of*.

RANK	CHEBI LABEL	CHEBI ID	PubMed Articles	q.value	odds-ratio	chisq-stat
1	phenylalanine	CHEBI:28044	3045	0	996.4	638213.3
2	5,6,7,8-tetrahydrobiopterin	CHEBI:15372	436	0	518.7	163713.7
3	tetrahydropterin	CHEBI:30436	440	0	485.6	156910.2
4	biopterin	CHEBI:15373	521	0	350.7	136557.8
5	biopterins	CHEBI:22881	524	0	342.6	134150.8
6	erythrose 4 - phosphate / phospho-enolpyruvate family amino acid	CHEBI:73690	3210	0	204.5	125691.2
7	aromatic amino acid	CHEBI:33856	3289	0	154.1	87093.1
8	amino acid zwitterion	CHEBI:35238	2930	0	110.0	83289.9
9	proteinogenic amino acid	CHEBI:83813	3336	0	61.7	32802.9
10	L-alpha-amino acid	CHEBI:15705	3383	0	56.0	27991.1
11	alpha-amino acid	CHEBI:33704	3404	0	51.7	25055.2
12	tyrosinate(1-)	CHEBI:32784	597	0	32.9	15455.9
13	tyrosine	CHEBI:18186	654	0	15.0	7078.0
14	L-alpha-amino acid anion	CHEBI:59814	629	0	14.5	6616.0
15	alpha-amino-acid anion	CHEBI:33558	629	0	14.5	6582.9
16	amino-acid anion	CHEBI:37022	629	0	14.5	6581.2
17	pterins	CHEBI:26375	563	0	15.2	6344.7
18	pteridines	CHEBI:26373	563	0	14.9	6194.7
19	diol	CHEBI:23824	441	7.03e-312	13.5	4518.9
20	polar amino acid	CHEBI:26167	894	5.02e-249	4.4	1846.7

Table S4: Top 20 hits of the FORUM search for the disease group Phenylketonurias (MeSH D010661) using propagation through the ChEBI ontology.

RANK	CHEMONT LABEL	CHEMONT ID	PubMed Articles	q.value	odds-ratio	Chisq_stat
1	Biopterins and derivatives	C0001651	553	0	250.3	107357.0
2	Pterins and derivatives	C0000110	593	0	16.6	7375.3
3	Tyrosine and derivatives	C0004319	718	0	14.1	7177.0
4	Pteridines and derivatives	C0000109	600	0	14.5	6394.8
5	Indolyl carboxylic acids and derivatives	C0001290	207	1.47e-110	8.4	1268.4
6	Phenylpyruvic acid derivatives	C0001276	62	2.08e-110	169.8	9321.2
7	Serotonins	C0001637	158	1.64e-36	3.5	265.5
8	Histidine and derivatives	C0004311	103	8.07e-36	4.9	309.7
9	Tryptamines and derivatives	C0000183	159	2.06e-34	3.3	244.4
10	Indole-3-acetic acid derivatives	C0001252	45	5.64e-23	7.6	246.1
11	Leucine and derivatives	C0004329	71	1.42e-17	3.6	127.4
12	2(hydroxyphenyl)acetic acids	C0004644	12	1.34e-14	41.1	420.8
13	Phenylacetic acids	C0000418	12	1.60e-14	40.5	414.0
14	Phenylpropanoic acids	C0002551	45	2.77e-14	4.4	112.6
15	Methionine and derivatives	C0004143	59	3.21e-14	3.5	100.6
16	Pyridoxines	C0001948	28	7.95e-14	7.1	138.7
17	Isoleucine and derivatives	C0004330	26	2.62e-11	6.0	102.2
18	Catecholamines and derivatives	C0000182	99	4.0e-08	1.9	42.6
19	D-alpha-amino acids	C0004145	33	2.55e-06	2.9	37.7
20	L-cysteine-S-conjugates	C0004555	19	2.97e-06	4.3	44.6

Table S5: Top 20 hits of the FORUM search for the disease group Phenylketonurias (MeSH D010661) using propagation through the ClassyFire (ChemOnt) ontology. The last two rows are not significant at the considered threshold.

S2 Supplementary Figures

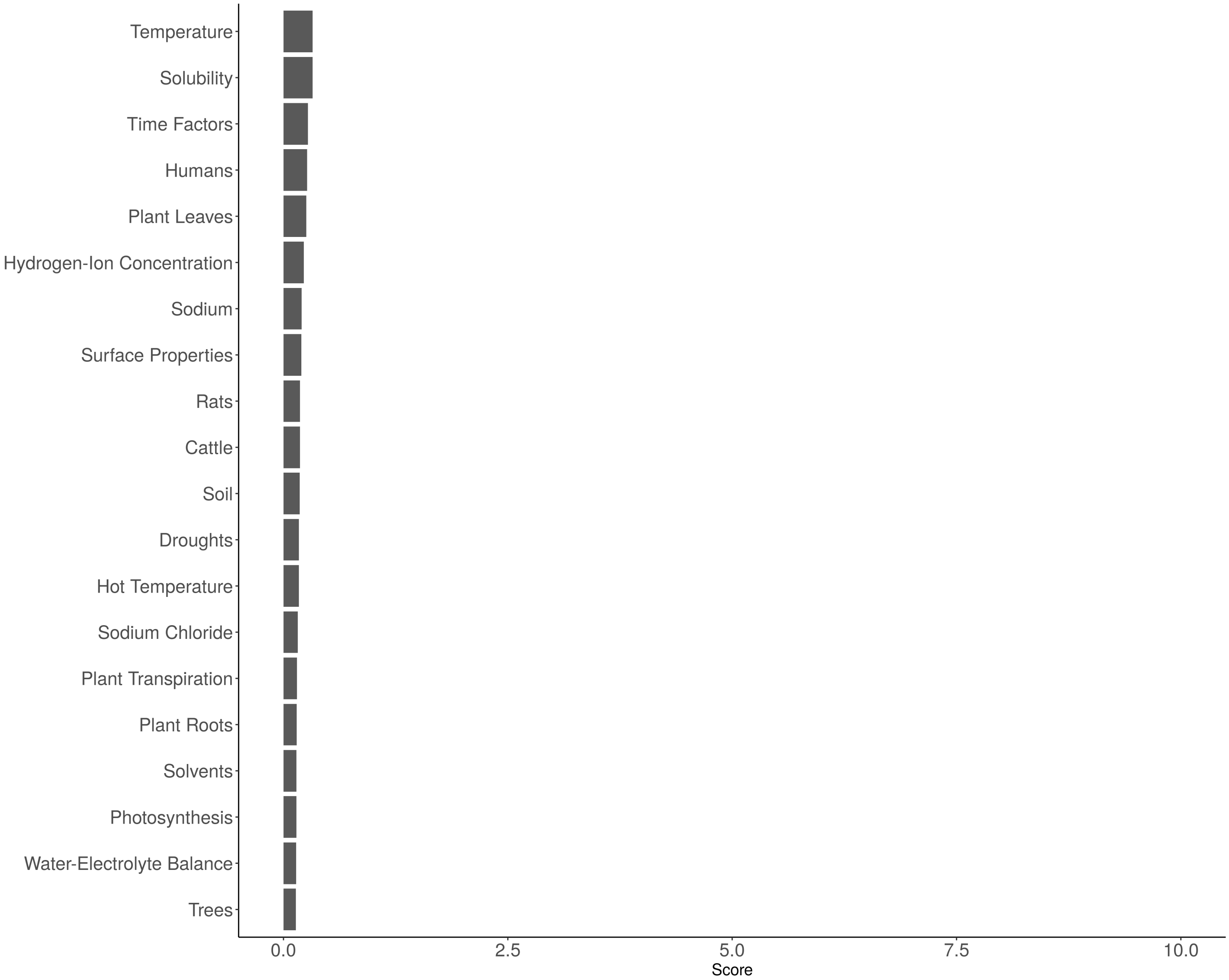

Figure S1: Top 20 most important descriptors describing the relation between Water and Eukaryote, according to the score proposed in S3.2.2. The MeSH descriptor associated with Water (*mesh:D014867*) was removed from the selection as it directly represents the studied compound.

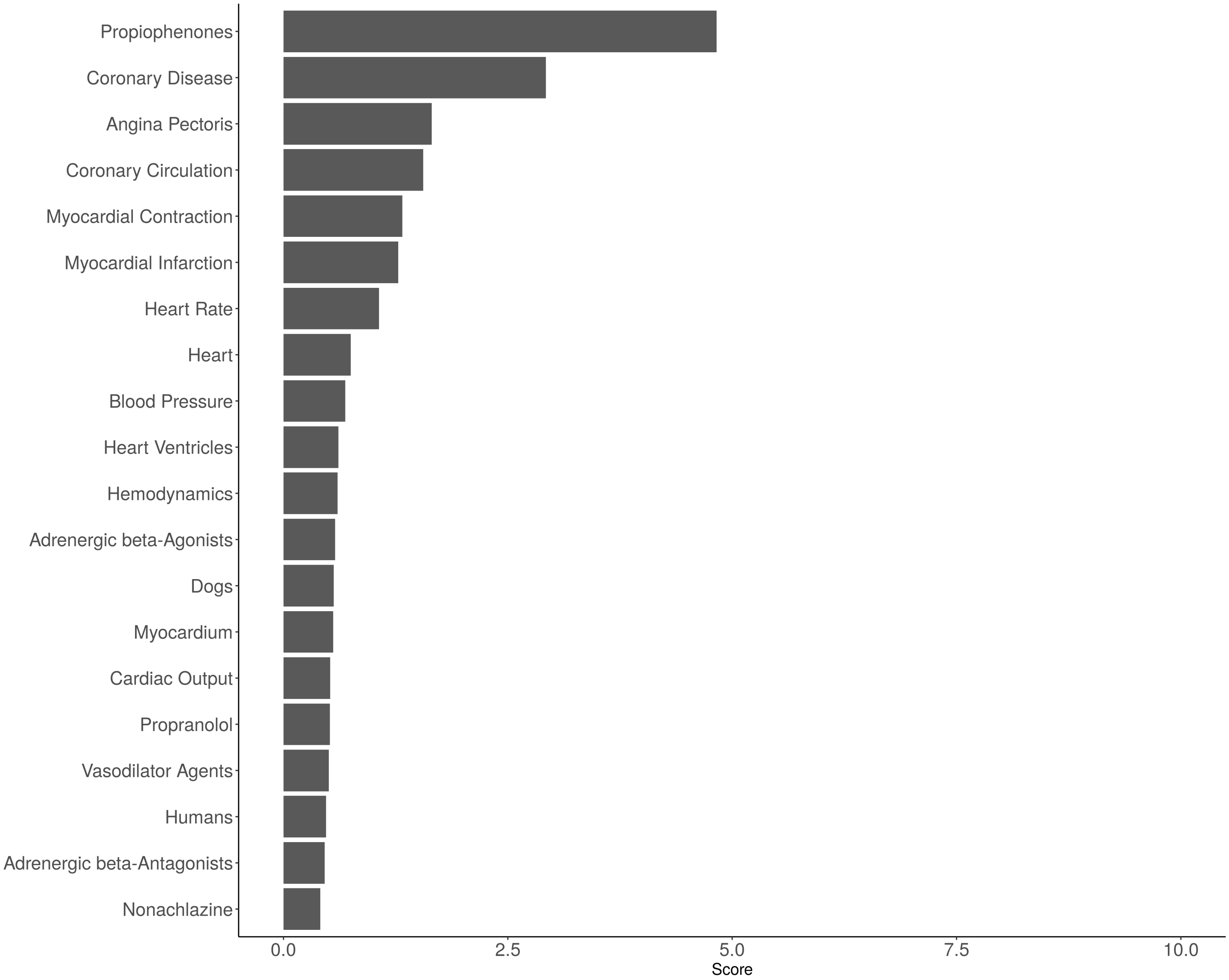

Figure S2: Top 20 most important descriptors describing the relation between Oxyfedrine and Myocardial Ischemia, according to the score proposed in S3.2.2. The MeSH descriptor associated with Oxyfedrine (*mesh:D010099*) was removed from the selection as it directly represents the studied compound.

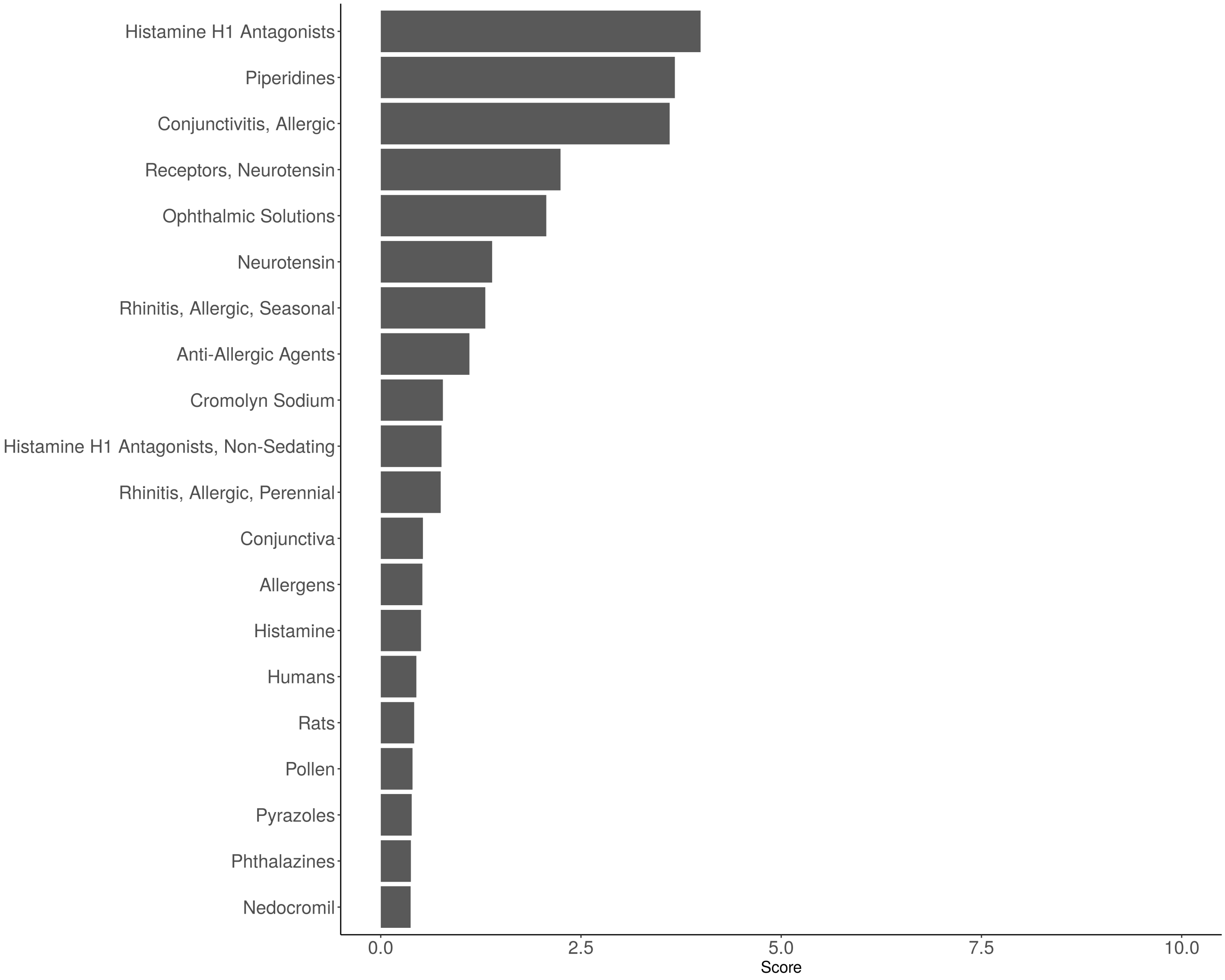

Figure S3: Top 20 most important descriptors describing the relation between Levocabastine and Eukaryote, according to the score proposed in S3.2.2.

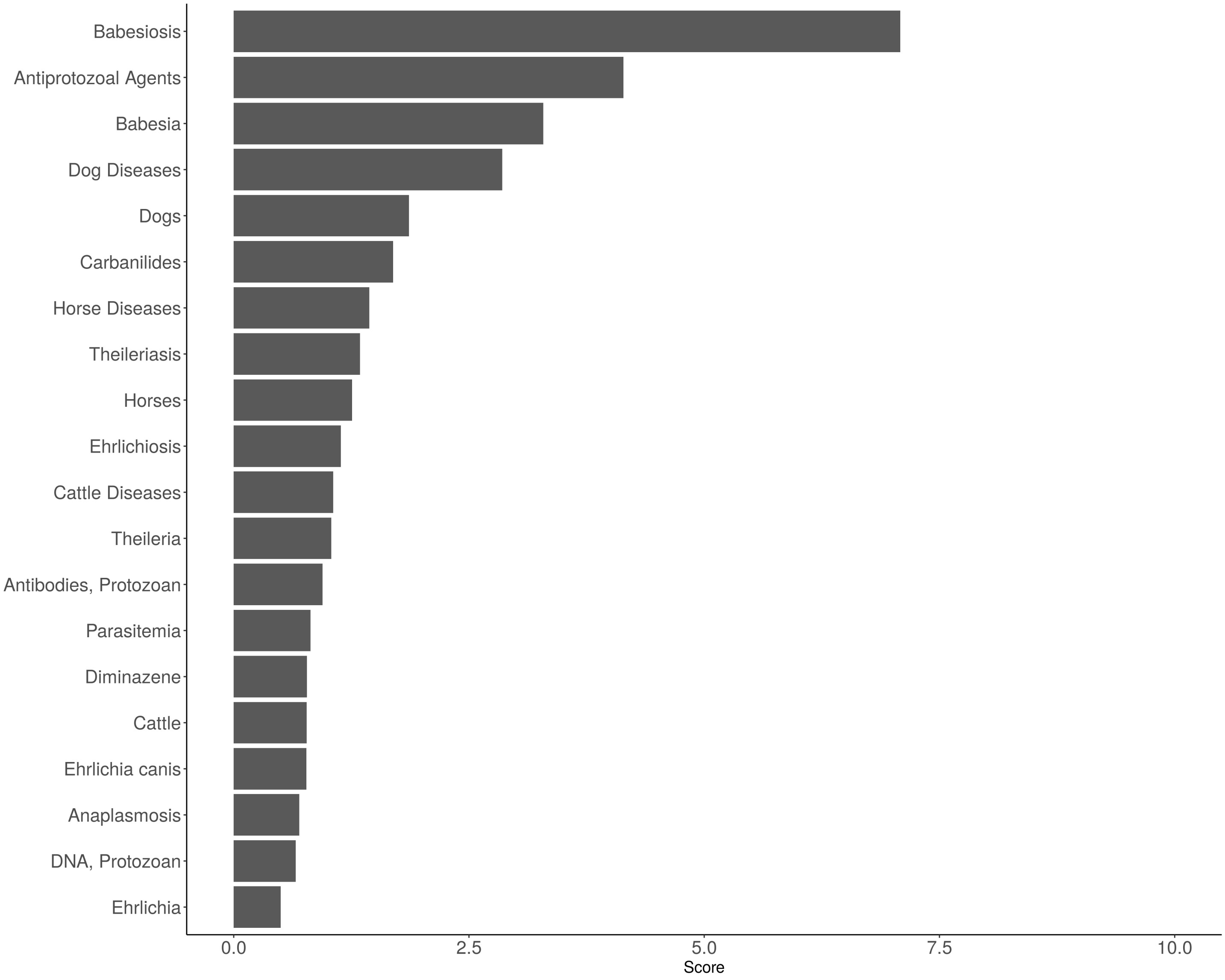

Figure S4: Top 20 most important descriptors describing the relation between Imidocarb and Tick diseases, according to the score proposed in S3.2.2. The MeSH descriptor associated with Imidocarb (*mesh:D007095*) was removed from the selection as it directly represents the studied compound.

S3 Supplementary material

S3.1 Supplementary Results: Impact of the semantic level on association extraction

S3.1.1 True-path rule impact on MeSH corpora size

The true-path rule modifies corpora sizes and thus co-occurrence counts used for statistical testing of independence. The impact of the true-path rule depends on the vocabulary structure, as a "flat" ontology would bring few changes. It also depends on the indexation practice, since overuse of the broadest descriptor would also lead to small changes. In order to explore the overall impact of the semantic level integration to our knowledge network, we checked how it influenced the corpus of MeSH descriptors and their extracted relations with PubChem compounds.

We first looked at the delta in corpus size associated with each MeSH descriptor when propagating, or not, the annotations through the MeSH Thesaurus, according to the *true-path* rule (Figure S5). We chose to organise each MeSH in categories by number of children, to observe the relation between the tree location and the potential benefit of the use of semantic relations. MeSH descriptors with no children ([0-1[) can be considered as leaves in the MeSH Tree, and the use of semantic relations has no impact on their corpus size, as there are no more specific terms in the Thesaurus from which to propagate the annotations. Benefits in terms of the number of added publications

increase with the hierarchical position of MeSH descriptors in the tree.

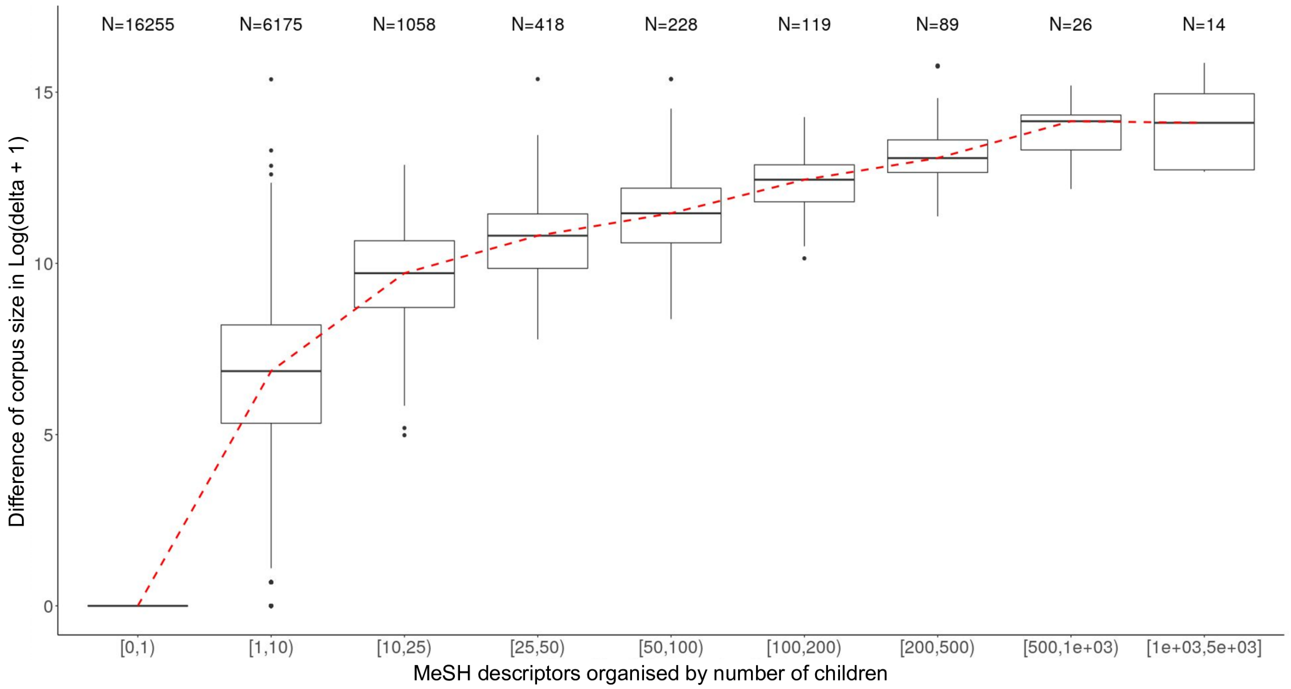

Figure S5: Comparison of the difference in corpus size associated with 24.382 MeSH descriptors with and without propagation through the MeSH Thesaurus. 117 MeSH originally have an empty corpus without propagation. MeSH are organised by number of children according to the MeSH tree, from leaves with no children [0-1[, like Gardner Syndrome (*mesh:D005736*), to root descriptors like Neoplasm (*mesh:D009369*). For each category, the number of MeSH descriptors that belong to it is indicated at the top of the boxplot. The red dashed line indicates the mean.

S3.1.2 True-path rule impact on association broadness

The MeSH corpus size distribution was compared between associations gained, discarded and kept (Figure S7). Corpus sizes were determined using the number of articles annotated to each MeSH descriptor after propagation according to the *true-path rule*. The *conserved* set is composed of 22,051 distinct MeSH descriptors ($\sim$ 97% of all studied MeSH with significant associations) and the *added* and *lost* sets are respectively composed of 7,622 and 7,769 MeSH descriptors. The distributions of MeSH corpus size are very similar for these last two as they share many descriptors in common. However, these descriptors are not specific to added or lost associations as they are also involved in conserved relations (Cf. Figure S6). Only 11 MeSH concepts aren't associated with any compounds after applying the true-path rule, such as: *Civilisation (D002962)* or *Archives (D001109)*. Also, 625 were added without having any associated compounds initially, like *Toxic Actions* (D004786). Finally, 59 MeSH are found both in the newly added associations and the lost ones, without being found in the conserved set, meaning that their associated compounds have completely changed. *Adnexal Diseases (D000291)* is an example of such changes. This term was originally rarely used for annotation (172 articles), resulting in few significant associations with compounds (only 2). However, its corpus has been heavily impacted by the true-path rule propagation (42,051 new articles added), since its narrower child concepts were much more used. This has diluted previous relations and revealed new ones from the new corpus.

Furthermore, there is a gap between the distribution of MeSH involved in conserved associations and those involved in added or lost associations (Cf. Figure S7). It seems that by using propagation, affected associations imply a subset of broader MeSH descriptors whose corpus are on average larger than those linked to conserved associations.

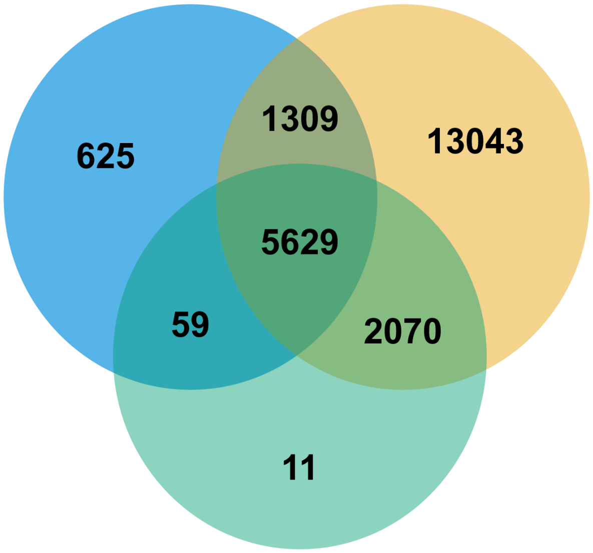

Figure S6: A Venn diagram of MeSH sets associated with conserved (orange), lost(green) or added (blue) associations. Among the 24,382 studied MeSH descriptors, 22,746 are involved in significant associations with PubChem compounds.

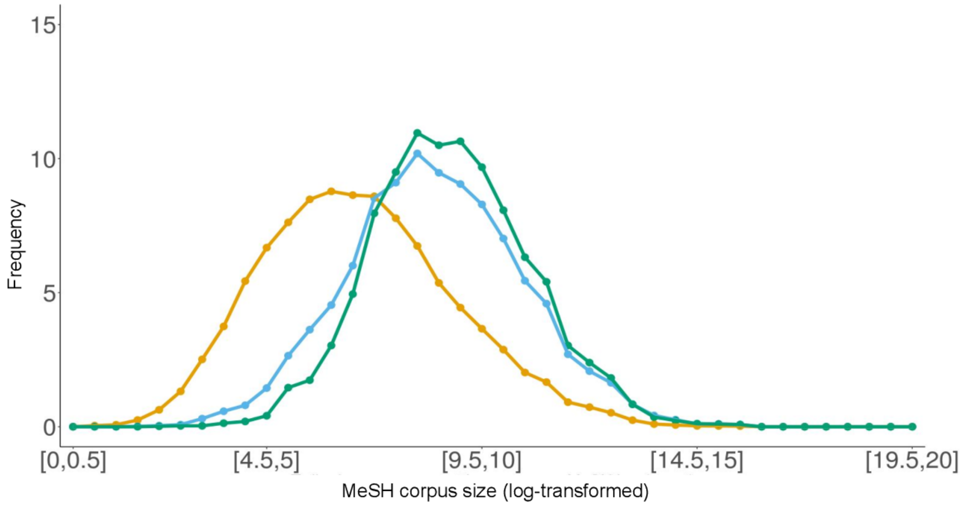

Figure S7: Corpus size distributions (Log-transformed) of MeSH sets associated with each category of the Venn diagram. The Corpus size of MeSH descriptors was determined with literature propagation through the MeSH Thesaurus. The colours are maintained from Figure 2 and S6: associations conserved (orange), lost (green) or added (blue).

S3.1.3 True-path rule impact on MeSH representation in associations

The impact of applying ontology based reasoning on extracted associations has been characterized considering MeSH tree positions. We first checked the distribution of significant associations among the MeSH trees by considering whether they are new, conserved or lost, Figure S8. For all MeSH categories, selected associations are in similar proportion compared to the whole set observed in the venn diagram ($\sim$ 50% of added, $\sim$ 47% conserved and $\sim$ 3% lost), except for the organisms category for which 63% of associations are new. See details on Supplementary table S6. Similar distributions are also found for subclasses of the Diseases MeSH tree (Figure S8). Differences in the absolute number of associations in each MeSH tree are directly linked to their global rate of annotation in the KG. As all our studied articles are retrieved from PubChem entry mentions, they might be more likely to be related to chemical compounds or activities, thus the *Chemicals & Drugs* tree is the most represented in our results ($\sim$ 52%). MeSH descriptors from *Infections* and *Neoplasm* sub-trees are likewise among the most annotated disease categories in PubMed.

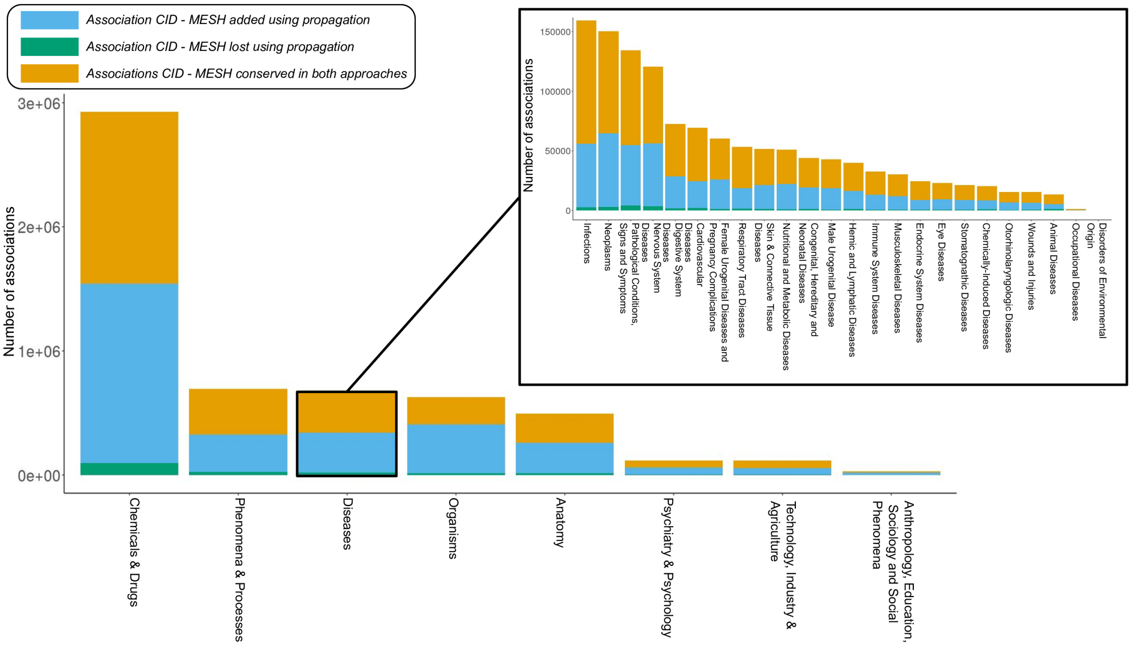

Figure S8: Stacked barplot describing the number of associations CID - MESH involved in each MeSH tree and organised by categories of the venn diagram (Cf. Figure 2). The distribution of associations in the Disease tree is also described in detail.

**Tree**	**N, Added**	**N, Lost**	**N, Conserved**	**% Added**	**% Lost**	**% Conserved**
Chemicals and Drugs	1446010	100705	1381315	49.38	3.44	47.17
Phenomena and Processes	301684	23912	366490	43.59	3.46	52.95
Diseases	320130	20475	337174	47.23	3.02	49.75
Organisms	393382	12547	220060	62.84	2.00	35.15
Anatomy	243582	14078	238380	49.11	2.84	48.01
Psychiatry and Psychology	53540	2886	59716	46.1	2.48	51.41
Technology, Industry, and Agriculture	58032	4143	54035	49.93	3.57	46.50
Anthropology, Education, Sociology, and Social Phenomena	16790	962	13663	53.45	3.06	43.50
**Global**	**2772905**	**173704**	**2581608**	**50.16**	**3.14**	**46.70**

Table S6: Number of added, lost and conserved associations with related percentages by MeSH trees. **Warning:** As one MeSH descriptor can be located in several different trees, counts for the Venn diagram do not have to be interpreted as the total.

S3.2 Supplementary Methods

S3.2.1 Fragility index

The *fragility index* aims at determining the number n of articles that, if removed from the corpus, would return a non significant *p-value*.

Due to the discrete nature of the data, when studying rare MeSH descriptors or compounds, having small corpora, small variations in the co-occurence can have a significant impact on results.

To determine n, several scenarios are estimated in which a growing number of articles supporting the relation between for instance, a compound (or a chemical class) A and a MeSH descriptor B would be removed. Nonetheless, not all scenarios can be tested for computational reasons and we need to establish bounds in which to determine scenarios. We used the Jeffrey interval (at 95%) to determine a confidence interval around the proportion of articles discussing a MeSH descriptor B among those discussing the compound A, $\frac{N_{ij}}{N_{i.}}$ with N_{ij} the number of articles discussing the MeSH j and the compound i (the co-occurrences) and $N_{i.}$ the total number of articles discussing the compound i.

As many compounds in the FORUM database are mentioned by only a few articles, we used Jeffrey confidence intervals, since they have the advantage of being appropriate for small sample sizes [42].

Using the lowest bound p_{min} of the interval, the co-occurrence is estimated by rounding N_{min}, corresponding to the scenario that led to such minimal proportion: $N_{min} \approx p_{min}N$.

This lowest scenario is used as an initial test: if the test is still significant, the association is declared robust and no other tests are computed, else, we compute all possible scenarios from N_{min} to the observed co-occurrence, to determine the first scenario that fails. Using this failing scenario, we determine n, the number of articles that if removed from the corpus, makes the *p-value* exceed the significance threshold ($1e - 6$). Because the *q-value* is always greater or equal to the *p-value*, this also informs us about the *q-value* that could be obtained from this scenario. See examples in Figures S9 and S10.

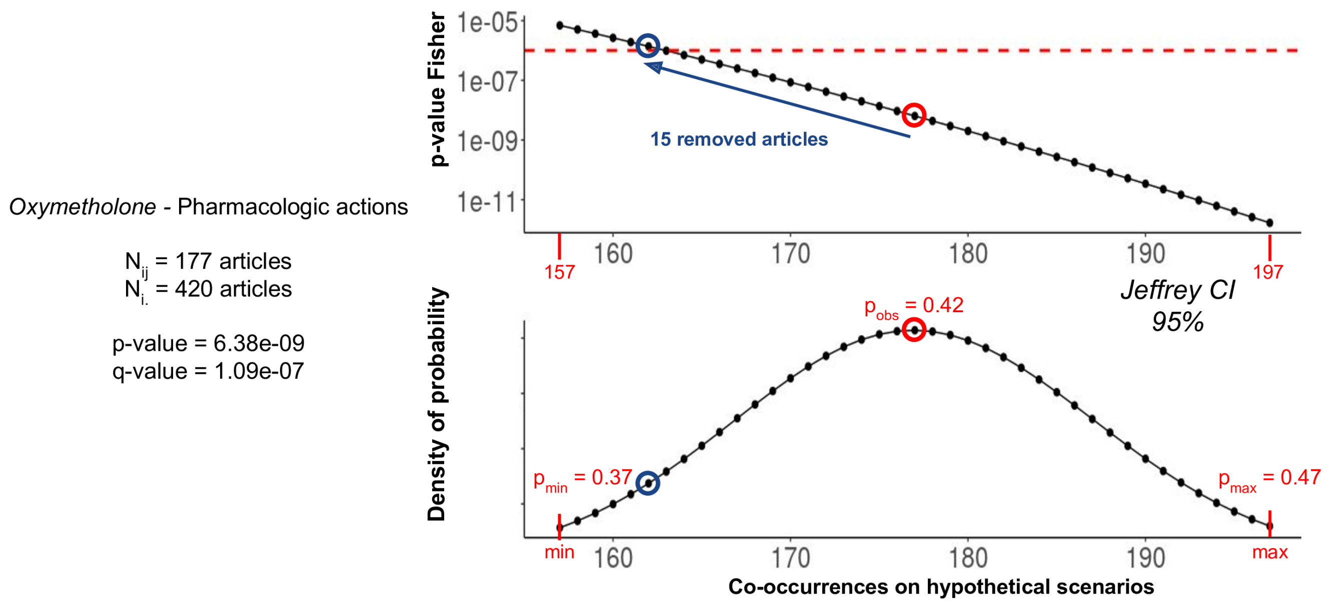

Figure S9: Example Oxymethone - Pharmacologic actions of the fragility index procedure

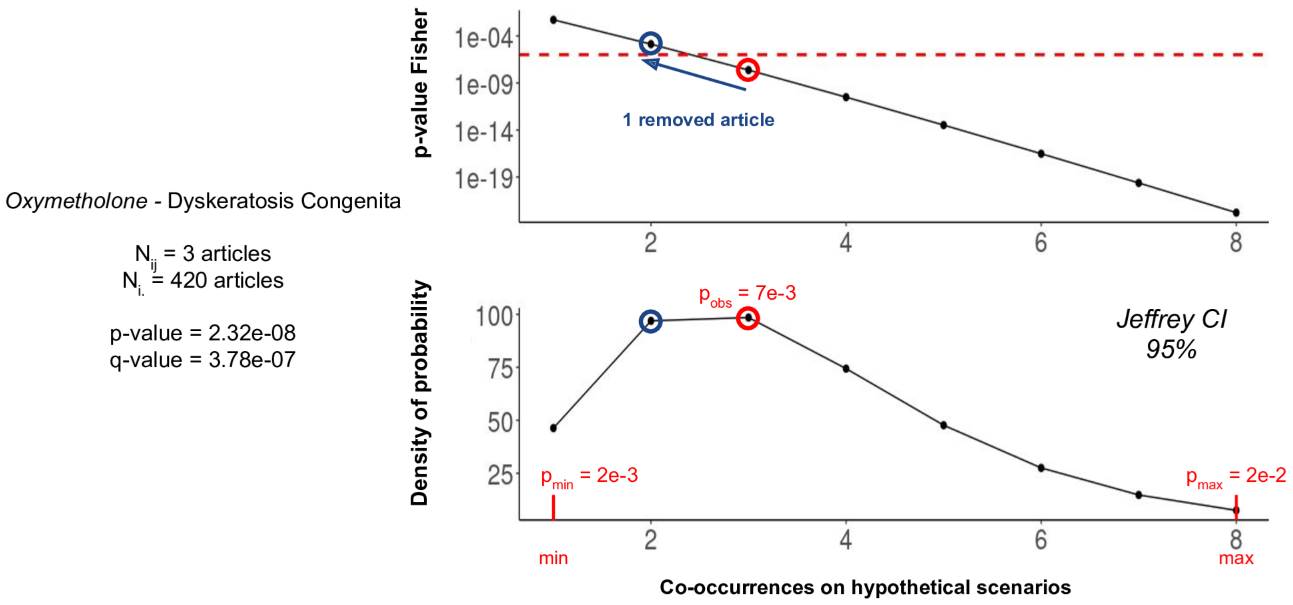

Figure S10: Example Oxymethone - Dyskeratosis Congenita of the fragility index procedure

The association between *Oxymethone* (an androgen and anabolic steroid) and *Pharmacologic actions* is supported by 177 articles, among 420 related to Oxymethone, corresponding to a proportion of ≈ 0.42. *Pharmacologic actions* is a broad descriptor with about 2.5 million articles. Using the lower bound of the Jeffrey CI on this proportion, we estimated the co-occurrence at the lowest scenario: 157 articles. Then, we computed each independence test between the lowest (157 articles) and the observed scenario (177 articles). From the obtained *p-values*, it appears that 15 articles should be removed from the association corpus to obtain a *p-value* higher than the considered threshold (and therefore also the q-value), and thus could change the decision on the relation relevance.

In a second example, we studied the relation between *Oxymethone* and *Dyskeratosis Congenita* (a X-linked recessive syndrome). This relation is only supported by 3 articles, but is nonetheless significant, notably because *Dyskeratosis Congenita* is rarely discussed in the KG (111 articles). Following the same procedure, we found that just by removing one article, this relation could no longer be significant.

The fragility index (FI) is meant to detect relations that require a manual examination by the user. If, for a FI of n, n publications in the relation supporting corpus seem irrelevant, the relation should be ignored. It is complementary to the *q-value* by giving a more formal interpretation of the strength of a relation.

In the previous example, two associations with *Oxymethone* have been flagged as *weak* from their Fragility Index. However, the number n of articles, on which the inclusion of the relation depends, is much higher in the first example (15 articles) than in the second (1 article), since it depends on the corpus size of the studied compound and MeSH. Because the supporting corpus for a widely discussed concept (e.g., pharmacologic actions) is larger than that for a less studied concept (e.g Dyskeratosis Congenita), we should remove more articles to exclude the relation. Thus, the value of the fragility index should be interpreted regarding the corpus size of the compound and the MeSH, and therefore should not be compared between relations.

S3.2.2 Score used for ranking co-annotated MeSH descriptors

To estimate the importance of each MeSH descriptor co-mentioned in the articles supporting a relation, we propose the computation of a score, analogous to the TF-IDF.

The TF-IDF (Term Frequency - Inverse Document Frequency) is a metric used in text-mining approaches to estimate the importance of a word in a document, regarding a collection of documents. We applied a similar methodology using terms in metadata (MeSH) rather than terms from text article body. Thus, the more frequently a descriptor is annotated in the articles supporting the relation compared to its overall annotation frequency, the higher the score.

To estimate the importance of a MeSH descriptor k annotated in publications supporting the relation between a compound i and a MeSH descriptor j:

$$Score = \frac{N_{i,j}^{k}}{N_{i,j}} \times log(\frac{N_{..}}{N_{.k}})$$

- $N_{i,j}$ the number of articles supporting the co-occurrence between the compound i and the MeSH descriptor j.
- $N_{i,j}^{k}$ the number of articles discussing the MeSH descriptor k among those supporting the relation between i and j.
- $N_{.k}$ The total number of articles discussing k in the KG.
- $N_{..}$ The total number of articles in the KG.

S3.3 Supplementary data: Swanson's Hypothesis - SPARQL requests

Here, we propose two SPARQL requests that can be used to retrieve links involved in Swanson's reasoning.

```
DEFINE input:inference "schema-inference-rules"
PREFIX rdf: <http://www.w3.org/1999/02/22-rdf-syntax-ns#>
PREFIX rdfs: <http://www.w3.org/2000/01/rdf-schema#>
PREFIX xsd: <http://www.w3.org/2001/XMLSchema#>
PREFIX owl: <http://www.w3.org/2002/07/owl#>
PREFIX meshv: <http://id.nlm.nih.gov/mesh/vocab#>
PREFIX mesh: <http://id.nlm.nih.gov/mesh/>
PREFIX voc: <http://myorg.com/voc/doc#>
PREFIX cito: <http://purl.org/spar/cito/>
PREFIX fabio:    <http://purl.org/spar/fabio/>
PREFIX owl: <http://www.w3.org/2002/07/owl#>
PREFIX void: <http://rdfs.org/ns/void#>
PREFIX cid:   <http://rdf.ncbi.nlm.nih.gov/pubchem/compound/>
PREFIX sio: <http://semanticscience.org/resource/>
PREFIX obo: <http://purl.obolibrary.org/obo/>
PREFIX skos: <http://www.w3.org/2004/02/skos/core#>
prefix dcterms: <http://purl.org/dc/terms/>
PREFIX chemont: <http://purl.obolibrary.org/obo/CHEMONTID_>

select distinct (strafter(STR(?other_compound),"http://rdf.ncbi.nlm.nih.gov/pubchem/compound/CID")
as ?CID)

from <https://forum.semantic-metabolomics.org/ClassyFire/direct-parent/2020>
from <https://forum.semantic-metabolomics.org/EnrichmentAnalysis/CID_MESH/2020>
from <https://forum.semantic-metabolomics.org/ChemOnt/2016-08-27>
from <https://forum.semantic-metabolomics.org/MeSHRDF/2020-12-07>
where
{
    {
        select ?other_compound
        where
        {
            mesh:D011928 skos:related ?RD_c .
            ?RD_c skos:related ?mesh_TU .
            ?mesh_TU (meshv:treeNumber|meshv:treeNumber/meshv:parentTreeNumber+) ?tn .
            ?mesh_selected meshv:treeNumber ?tn .
            VALUES ?mesh_selected { mesh:D002317 mesh:D006401 }
            ?other_compound skos:related ?mesh_TU .
            ?other_compound skos:related mesh:D005395 .
            ?other_compound a chemont:0003909 .
            }
        }
    FILTER NOT EXISTS {?other_compound skos:related mesh:D011928}
}
```

In this requests, we extracted:

- Compounds related to the Raynaud's Disease (mesh:D011928)
- MeSH descriptors significantly related to these compounds, by selecting those that are related to therapeutic actions: Cardiovascular Agents or Hematologic Agents.
- We then retrieve other compounds related to these MeSH descriptors
- We also specify that these new compounds must :

– be related to fish oils (mesh:D005395)
– belong to the chemical class of Fatty Acyls (chemont:0003909).
– not be already related to Raynaud's Disease in our KG.

Therefore, these Fatty Acyls compounds associated with fish oils are related to the same therapeutic effects (Cardiovascular Agents or Hematologic Agents) as some compounds used as treatment for Raynaud's Disease. Here we retrieve for instance Eicosapentaenoic acid (EPA), which indeed have the same therapeutic effect (Platelet Aggregation Inhibitors) as PGE1 or PGI2 which are used to treat Raynaud's disease.

```
DEFINE input:inference "schema-inference-rules"
PREFIX rdf: <http://www.w3.org/1999/02/22-rdf-syntax-ns#>
PREFIX rdfs: <http://www.w3.org/2000/01/rdf-schema#>
PREFIX xsd: <http://www.w3.org/2001/XMLSchema#>
PREFIX owl: <http://www.w3.org/2002/07/owl#>
PREFIX meshv: <http://id.nlm.nih.gov/mesh/vocab#>
PREFIX mesh: <http://id.nlm.nih.gov/mesh/>
PREFIX voc: <http://myorg.com/voc/doc#>
PREFIX cito: <http://purl.org/spar/cito/>
PREFIX fabio: <http://purl.org/spar/fabio/>
PREFIX owl: <http://www.w3.org/2002/07/owl#>
PREFIX void: <http://rdfs.org/ns/void#>
PREFIX cid: <http://rdf.ncbi.nlm.nih.gov/pubchem/compound/>
PREFIX sio: <http://semanticscience.org/resource/>
PREFIX obo: <http://purl.obolibrary.org/obo/>
PREFIX skos: <http://www.w3.org/2004/02/skos/core#>
prefix dcterms: <http://purl.org/dc/terms/>
PREFIX chemont: <http://purl.obolibrary.org/obo/CHEMONTID_>

select distinct (strafter(STR(?other_compound),"http://rdf.ncbi.nlm.nih.gov/pubchem/compound/CID")
as ?CID)
from <https://forum.semantic-metabolomics.org/EnrichmentAnalysis/CID_MESH/2020>
from <https://forum.semantic-metabolomics.org/MeSHRDF/2020-12-07>
where
{
        {
                select distinct ?other_compound
                from <https://forum.semantic-metabolomics.org/EnrichmentAnalysis/CID_MESH/2020>
                from <https://forum.semantic-metabolomics.org/ClassyFire/direct-parent/2020>
                where
                {
                        {
                                select ?RD_class
                                from <https://forum.semantic-metabolomics.org/EnrichmentAnalysis/
                                CHEMONT_MESH/2020>
                                from <https://forum.semantic-metabolomics.org/MeSHRDF/2020-12-07>
                                where
                                {
                                        mesh:D011928 skos:related ?RD_class .
                                        ?RD_class skos:related ?mesh_TU .
                                        ?mesh_TU (meshv:treeNumber|meshv:treeNumber/
                                        meshv:parentTreeNumber+) ?tn .
                                        ?mesh_selected meshv:treeNumber ?tn .
                                        VALUES ?mesh_selected { mesh:D002317 mesh:D006401 }
                                }
                        }
                ?other_compound a ?RD_class .
                ?other_compound skos:related mesh:D005395 .
```

```
                FILTER NOT EXISTS {?other_compound skos:related mesh:D011928}
                }
        }
        FILTER NOT EXISTS {
                ?other_compound skos:related ?mesh_TU .
                ?mesh_selected meshv:treeNumber ?tn .
                VALUES ?mesh_selected { mesh:D002317 mesh:D006401 }
                ?mesh_TU (meshv:treeNumber|meshv:treeNumber/meshv:parentTreeNumber+) ?tn
        }

}
```

In this request, we extracted:

- Chemical classes related to Raynaud's Disease which are also related to therapeutic actions: Cardiovascular Agents or Hematologic Agents
- We then selected other members of these chemical classes that are related to fish oils but not to Raynaud's Disease.
- We also specify these compounds must **not** be associated with therapeutic effects (Cardiovascular Agents or Hematologic Agents) in our KG.

These are therefore compounds associated with fish oils that belong to the same chemical class as Raynaud's disease-related compounds with therapeutic effects (cardiovascular agents or haematological agents), but for which no such effects are reported. This allows us to retrieve for instance PGI3, the analog of PGI2, which is used as treatment of the Raynaud's diseases for its properties of vasodilator and antiplatelet agents.
